# Using New Vaginal Doses Evaluation System to Assess the Dose–Effect Relationship for Vaginal Stenosis After Definitive Radio(Chemo)Therapy for Cervical Cancer

**DOI:** 10.3389/fonc.2022.840144

**Published:** 2022-04-19

**Authors:** Juan Wang, Kai-shuo Zhang, Zi Liu, Tao Wang, Rui-hua Wang, Fu-quan Zhang, Lang Yu, Ya-li Wang, Li-chun Wei, Mei Shi, Sha Li, Bao-gang Liu, Fan Shi, Jin Su, Wei Yuan, Qi ying Zhang, Jing Zhang

**Affiliations:** ^1^ Department of Radiation Oncology, The First Affiliated Hospital of Xi’an Jiao Tong University, Xi’an, China; ^2^ Department of Radiation Oncology, Hanzhong Center Hospital, Hanzhong, China; ^3^ Department of Radiation Oncology, Peking Union Medical College Hospital, Chinese Academy of Medical Sciences and Peking Union Medical College, Beijing, China; ^4^ Department of Radiation Oncology, The Second Affiliated hospital of Xi’an Jiao Tong University, Xi’an, China; ^5^ Department of Radiation Oncology, First Affiliated Hospital of Air Force Medical University, Xi’an, China; ^6^ Department of Radiation Oncology, The 940th Hospital of Joint Logistics Support force of Chinese People’s Liberation Army, Lanzhou, China; ^7^ Department of Radiation Oncology, The Second Affiliated Hospital of Shaanxi University of Chinese Medicine, Xianyang, China

**Keywords:** radiotherapy, PIBS points system, ICRU rectum point, vaginal stenosis, cervical cancer

## Abstract

**Objective:**

The study aims to investigate if a relationship exists between vaginal doses and vaginal stenosis (VS) using posterior–inferior border of symphysis (PIBS) points and the International Commission on Radiation Units-Rectum (ICRU-R) point evaluation system for definitive radio(chemo)therapy in locally advanced cervical cancer.

**Methods and Materials:**

From a vaginal dose study in China, 351 patients were prospectively assessed. For every reference point of the PIBS system and ICRU-R point was calculated for all BT and summed with EBRT. Pearson’s chi-square test and Student’s unpaired t-test compared variables with and without vaginal stenosis (VS) G ≥2. The risk factors were assessed for VS G ≥2 in multi- and univariate analyses through Cox proportional hazards model followed by a dose–effect curve construction. The VS morbidity rate was compared *via* the log-rank test using the median vaginal reference length (VRL).

**Results:**

The patients (38-month median follow-up) had 21.3% three-year actuarial estimate for VS G ≥2. Compared to G <2 patients, VS G ≥2 patients received higher doses to PIBS points except for PIBS − 2 and had significantly shorter VRL. VRL (HR = 1.765, P = 0.038), total EBRT and BT ICRU-R point dose (HR = 1.017, p = 0.003) were risk factors for VS. With VRL >4.6 cm, the 3-year actuarial estimate was 12.8% vs. 29.6% for VRL ≤4.6 cm. According to the model curve, the risks were 21, 30, and 39% at 75, 85, and 95 Gy, respectively (ICRU-R point dose).

**Conclusions:**

PIBS system point doses correlated with late vaginal toxicity. VRL combined with both EBRT and BT dose to the ICRU-R point contribute to VS risk.

## Introduction

The vagina is a target organ and organ at risk (OAR) in radiation therapy for cervical cancer (CC). To date, morbidity rates due to vaginal conditions for grades II and III or higher vaginal stenosis (VS) in patients of CC treated with radio(chemo)therapy were 17–41.7 and 1–17.6% respectively (1–5). VS is commonly defined as the loss of elasticity and mucosal atrophy, shortening and narrowing of the vaginal canal, and pain during exam/intercourse (6, 7). It can seriously worsen the quality of life of patients and even recurrent tumors cannot be found due to inability to open a vaginal speculum. A few studies have revealed that the dose of ICRU-R point and D2cc in the vagina as the predictors of VS occurring after pelvic radiotherapy, besides, is no clear specification regarding accepted dosimetry, vaginal radiation tolerance, and the limitations of the vagina as an OAR (8).

The PIBS point system proposed for the first time at the Medical University, Vienna is a newly defined method of vaginal dose-reporting for both EBRT (external beam radiotherapy) and BT (brachytherapy) (9). This method contains different points total (BT + EBRT) dose administered to the lower, upper, and middle vaginal parts. This system of evaluation can be applied for 2D/3D BT. We earlier studied whether this method can be applied in patients, and found that Chinese patients with a shorter median vaginal reference length (VRL) of <4.5 cm received relatively higher doses of radiation at the PIBS − 2 cm, PIBS, and PIBS + 2 cm points than those of American and European patients (10). In this study, we further investigated if a relationship exists between vaginal doses and VS using PIBS points and ICRU-R point evaluation system in locally advanced CC treated with definitive radio(chemo)therapy.

## Methods

### Patient Characteristics, Treatment Modality, and Endpoint Assessment

Three hundred fifty-one patients with cervical cancer FIGO stage IB–IVB except IIIA were recruited between December 2016 and June 2018 from six radiation centers in China with locally advanced CC participated in the clinical trial (NCT03257475) and were treated with 45–50 Gy 3D-conformal radiotherapy or IMRT (intensity-modulated radiotherapy), with weekly simultaneous administration of 25 mg/m^2^ cisplatin (11), or a combination of 25 mg/m^2^ cisplatin (days 1–3) with 135 mg/m^2^ liposome paclitaxel (day 1). High dose rate BT with two T&O implants were conducted separately, was administered doses of 24 or 30 Gy in four to five fractions. For treatment, the method of point A dose-evaluation was applied, and the dose of loading was according to the Manchester system of BT. Doses were reported in total EBRT and BT EQD2 dose using the linear quadratic model (α/β = 3 Gy for vaginal, α/β = 10 Gy for tumor) (12).

VS was scored in accordance with the CTCAE v3.0 (Common Terminology Criteria for Adverse Events v3.0) and prospectively assessed at baseline by the physician, at three months interval after the treatment ended in the first two years and at six months interval in the years three to five, and thereafter, annually. As per CTCAE v3.0, the definition of G1 is narrowing and/or shortening but not causing functional interference, G2 as narrowing and/or shortening along with functional impairment, and G3 as absolute vaginal obliteration and unmenable to correction by surgery (5). Here, the definition of primary outcome was the time from the completion of radiotherapy to first G ≥2 VS occurrence before local recurrence or death. Censored patients were those with no documented G ≥2 VS before recurrence for the first time or death on the date of the first relapse or last visit.

### Data Collection and Definitions

All reference points of the PIBS system proposed by the Westerveld group have been defined in our previous study (8). ICRU-R and ICRU-B points were also done based on the ICRU guidelines (Report 89) and the ICRU (Report 38) (13, 14). The ICRU-R point is associated with the applicator and positioned 5 mm on an anterior–posterior line at the back of the posterior vaginal wall drawn from the center of the vaginal source. The dose of A point, PIBS, ICRU-R, PIBS ± 2 cm, and ICRU-B points were recorded. Meanwhile, the value of VRL and other clinical data of patients were all obtained. A CT scan of patients was done at the time of the first and third fractions (BT1, BT3). The parameters of BT2, 4, and 5 were recorded by averaging the BT1 and BT3 dose values.

### Statistical Analysis

This study identified a set of variables in the dosimetric and clinical data for predicting of VS G ≥2 occurrence. Based on the distribution, data were presented as mean and SD (standard deviation) or median and IQR (interquartile range). Proportions were presented as the percentage of patients without and with the characteristic. The differentiation of continuous variables was done by student’s (unpaired) t-test and the comparison of categorical variables was done using Pearson’s chi-square test. Several *a priori* chosen, clinically relevant characteristics of disease-, patient-, and treatment were assessed as risk factors for VS G ≥2 in uni- and multivariate analyses by Cox regression model. Ninety-five percent CI (confidence intervals) and HR (Hazard Ratios) were estimated. Analysis of data at the time of the event was done by Kaplan–Meier analyses. The level of two-sided significance was set at 5%. Analyses of statistical data were conducted using SPSS v 23 (SPSS Inc., USA).

## Results

**Figure 1:**
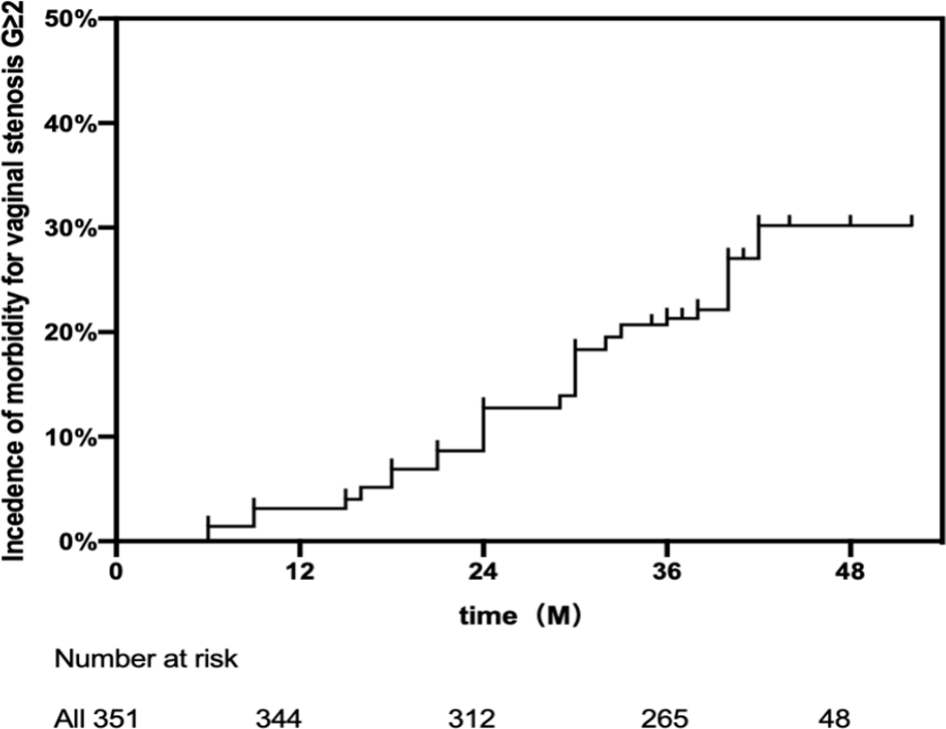
Cumulative incidence for vaginal stenosis G ≥2.

For this study, the time for a median follow-up was three years and two months (IQR 36–42 months). The disease, demography, and treatment characteristics of 351 patients are shown in **Table 1**. The median age was 50 years (range 31–60 years). Most patients (64.7%) had stages IIB (227/351) or IIIB (63/351, 17.9%) lesions. At the time of diagnosis, among 351 patients, 52 (14.8%) had a vaginal involvement according to clinical assessment and imaging tests. Most lesions were observed to be confined to the fornix of the vagina. The median VRL was 4.6 (IQR 3.8–5.2) cm. The doses of median total (BT + EBRT) at PIBS, PIBS − 2 cm, and PIBS + 2 cm were 55.8 (IQR 54.2–59.3) Gy_3_, 2.4 (IQR 2–3.1) Gy_3_, and 80.9 (IQR 65.8–102.8) Gy_3_, respectively. At point A, the median dose administered was 84.1 (IQR 81–87.5) Gy_10_. In 44 patients (12.5%), vaginal stenosis was not found (G0); 213 patients were observed to have G1 (60.6%); 90 G2 (25.6%) and 4 G3 (1.1%). The 3-year actual estimate VS G ≥2 was 21.31% (**Figure 1**).

**Table 1:** Patient, disease, and treatment characteristics.

Patient and disease characteristics	N = 351		
Median follow-up time	Median in months (IQR)	27	25–31
Age	Median in years (range)	50	31–60
FIGO stage (n, %)	IB	18	5.1
	IIA	32	9.1
	IIB	227	64.7
	IIIB	63	17.9
	IVB	11	3.1
Histology	Squamous cell carcinoma	348	99.1
	Adenocarcinoma	3	0.9
Tumor extension in the vagina at time of diagnosis (n, %)	Not involved	299	85.2
	Upper part	52	14.8
Treatment characteristics			
EBRT dose	Median dose in Gy (IQR)	50	45–50
EBRT technique (n, %)	3D-CRT	84	24
	IMRT	267	76
VRL	Median in cm (IQR)	4.6	3.8–5.2
EBRT+ BT point A in EQD2_10_	Median dose in Gy (IQR)	84.1	81–87.5
Dose to the PIBS reference point in EQD2_3_	Median dose in Gy (IQR)	55.8	54.2–59.3
Dose to the PIBS + 2 reference point in EQD2_3_	Median dose in Gy (IQR)	80.9	65.8–102.8
Dose to the PIBS − 2 reference point in EQD2_3_	Median dose in Gy (IQR)	2.4	2–3.1
Dose to the ICRU-R reference point in EQD2_3_	Median dose in Gy (IQR)	75.1	67.2–86.3
Dose to the ICRU-B Reference point in EQD2_3_	Median dose in Gy (IQR)	73.7	68.2–82.1

Proportions are presented as the number of total and percentages, continuous variables are given with median and interquartile range.

An analysis of factors as per the morbidity groups revealed different vaginal doses and VRL in two groups (**Table 2**). The results show that doses delivered to PIBS + 2, PIBS, and PIBS − 2 were higher (108.1 ± 55.4 Gy vs. 90.2 ± 40.8 Gy, P = 0.0059, 59.49 ± 7.1 6 vs. 57.8 ± 7. P = 0.067 and 2.8 ± 1.2vs. 2.6 ± 1.0 Gy P = 0.099) for patients having VS G ≥2 compared to those delivered in patients with G <2. Likewise, doses delivered to ICRU-R and ICRU-B points were significantly larger (85.8 ± 18.1 Gy vs. 75.7 ± 12.47Gy, P <0.0001 and 83.2 ± 13.6Gy vs. 76.1 ± 12.4 Gy, P = 0.007) for patients with VS G ≥2 compared with patients with G <2. However, values of VRL were significantly shorter for patients with VS G ≥2 compared to those in patients having G <2 (4.25 ± 0.99 cm vs. 4.69 ± 0.97 cm, P = 0.0003).

**Table 2:** A comparison of risk factors for vaginal stenosis G ≥2 to <2.

	VS <2	VS ≧2	P	χ^2^
Age(years)	49.5 ± 7.1 (31–64)	51.5 ± 6.3 (40–63)	0.015	–
BMI	23.9 ± 4.1	24.0 ± 4.0	0.827	–
Tumor extension in the vagina at time of diagnosis(n)				
Not involved	238 (79.6)	61 (20.4)	0.66	0.193
Upper part	40 (76.9)	12 (23.1)		
VRL(n)(%)				
≤4.6 cm	114 (64.4)	63 (35.6)	0.00001	19.710
>4.6 cm	148 (85.1)	26 (14.9)		
BT A point in EQD2_10_(Gy)	81.7 ± 0.4	81.7 ± 0.9	0.486	–
Dose to the PIBS reference point in EQD2_3_(Gy)	57.8 ± 7.6	59.49 ± 7.1	0.067	–
Dose to the PIBS + 2 reference point in EQD2_3_(Gy)	90.2 ± 40.8	108.1 ± 55.4	0.0059	–
Dose to the PIBS − 2 reference point in EQD2_3_(Gy)	2.6 ± 1.0	2.8 ± 1.2	0.099	–
Dose to the ICRU-R reference point in EQD2_3_(Gy)	75.7 ± 12.47	85.8 ± 18.1	<0.0001	–
Dose to the ICRU-B eference point in EQD2_3_(Gy)	76.1 ± 12.4	83.2 ± 13.6	0.007	–

Furthermore, in the multivariable model, VS risk factors related to an increase in the risk of G ≥2 VS were the following: values of VRL (binary, ≤4.6 cm vs. >4.6 cm, HR = 1.765, 95% CI 1.033–3.016, P = 0.038), and total EBRT and BT ICRU-R point dose (continuous EQD_2_ in Gy, HR = 1.017, 95% CI 1.006–1.028, p = 0.003). An overview of the uni- and multivariate Cox regression model is presented in **Table 3**.

**Table 3:** Univariate and multivariate analyses of risk factors for vaginal stenosis G ≥2.

N=351	Univariate Cox regression, p-value	Univariate Cox regression, Hazard Ratio [95% CI]	Multivariable Cox regression, p-value	Multivariable Cox Regression, Hazard Ratio [95% CI]
Age (continuous)	0.167	1.022 (0.991–1.055)	–	–
BMI (continuous)	0.673	0.989 (0.941–1.040)	0.928	1.003 (0.948–1.060)
FIGO Stage	0.354	1.119 (0.882–1.421)	–	–
Tumor extension in the vagina at time of diagnosis((binary)	0.078	1.135 (1.078–1.191)	–	–
VRL(binary ≤4.6 cm vs. >4.6 cm)	≤0.001	2.954 (1.711–5.101)	0.038	1.765 (1.033–3.016)
BT A point in EQD2_10_	0.483	1.109 (0.831–1.481)	–	–
Dose to the PIBS reference point in EQD2_3_(continuous)	0.051	1.024 (1.000–1.048)	–	–
Dose to the PIBS + 2 reference point in EQD2_3_ (continuous)	0.002	1.006 (1.002–1.009)	0.482	1.002 (0.997–1.007)
Dose to the PIBS − 2 reference point in EQD2_3_ (continuous)	0.106	1.153 (0.970–1.372)	–	–
Dose to the ICRU-R reference point in EQD2_3_ (continuous)	≤0.001	1.021 (1.012–1.032)	0.003	1.017 (1.006–1.028)
Dose to the ICRU-B reference point in EQD2_3_ (continuous)	0.002	1.014 (1.005–1.023)	0.922	1.001 (0.948–1.060)

**Figure 2:**
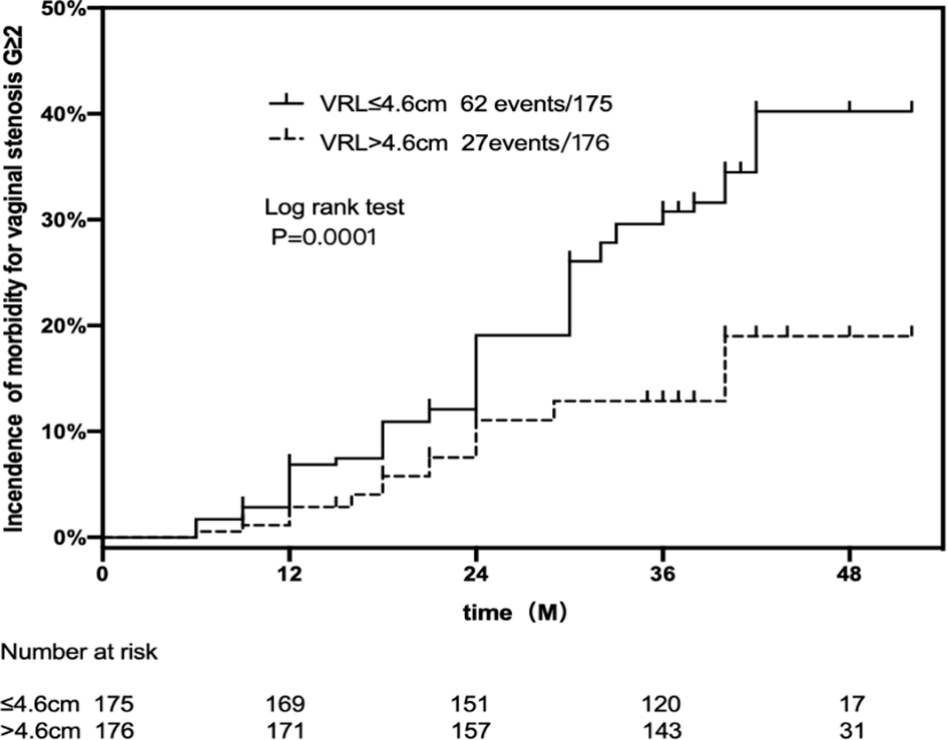
Actuarial estimates for VS G ≥2 in patients VRL ≤4.6 cm in comparison to those with VRL >4.6 cm.

In VS G ≥2 group, most patients (69.7%) had VRL of ≤4.6 cm, while only 30.3% had VRL >4.6 cm. The probability of occurrence of VS G ≥2 according to this length cut-off is mentioned in **Figure 2**. With longer VRL (>4.6 cm), the three-year actuarial estimate was 12.8%, while with shorter VRL (≤4.6 cm), the value was 29.6%.

**Figure 3:**
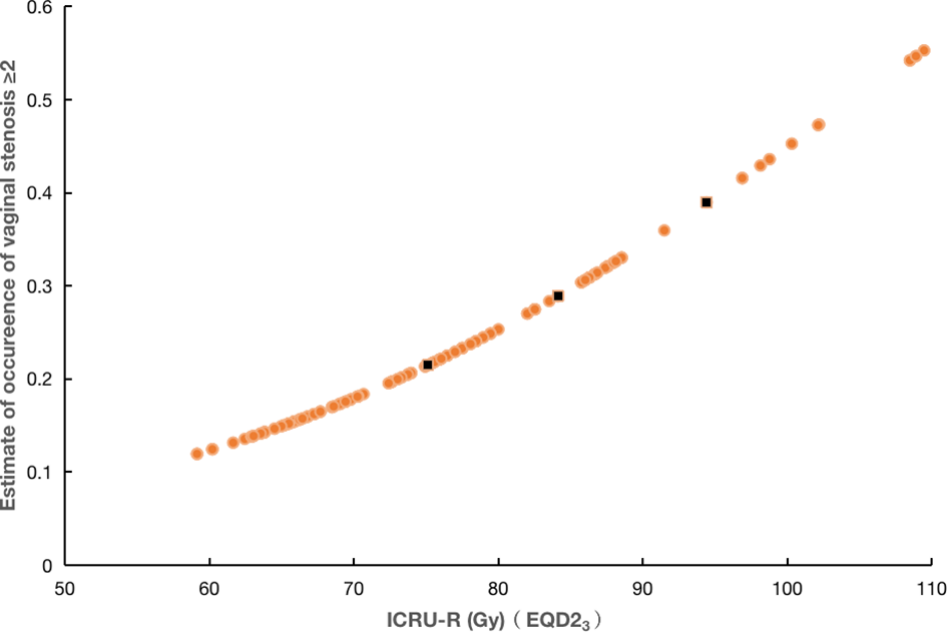
The relationship of dose–effect of the combined brachytherapy and EBRT dose to ICRU-R point in EQD2 and VS G≥2 in (N = 351) patients. Three black points represent ICRU-R point doses of 75, 85, and 95 Gy, respectively.

In the relationship between dose–effect of VS with an increase in the dose to the ICRU-R point, there was a significant increase in the probability of VS G ≥2 to increase (p <0.001). A dose–effect curve according to the risk determined by univariate Cox regression test is shown in **Figure 3** with estimation for VS G ≥2 and ICRU-R point dose in EQD_2_ (HR = 1.021 per 1 Gy with 95% CI 1.012–1.032). As per the model for dose–effect, the probability to develop VS G ≥2 were 21, 30, and 39% with ICRU-R point doses of 75, 85, and 95 Gy, respectively.

## Discussion

In this multi-center, prospective study using the PIBS points and ICRU-R point evaluation system, we found VRL and ICRU-R point-dose (total EBRT + BT) as two risk factors for VS G ≥2 in CC post-definitive radio(chemo)therapy. With shorter VRL and an increase in dose to the ICRU-R point, there was a significant increase in the probability of the occurrence of VS.

In this study, most patients received IMRT + 2D BT and the median dose of point A in EQD2 was 84.1 Gy. Although three-dimensional and IGABT (image-guided adaptive brachytherapy) has been recommended by EMBRACE study (15), many hospitals around the world cannot promote this technology due to the limitations of equipment and personnel, so the two-dimensional and A point-dose evaluation system is still an important method in BT of cervical cancer. Furthermore, the rate of morbidity due to VS after definitive radiotherapy was similar to that obtained in other studies that applied IGABT. Severe or no morbidity VS is rare, and most studies have reported mild-to-moderate morbidity (3, 16–19). In a few earlier retrospective studies, patterns of standard loading directed to point A was used and attempted to define a maximum vaginal mucosa tolerance dose. Most of these reports on morbidity due to vaginal side effects are from mono-institutional, retrospective studies and have used varying systems for side-effect grading, and therefore, cannot be used for comparing with the results of this study.

As far as we are aware, this report is the first on the use of the PIBS system to evaluate the dose–effect of VS in patients with CC. The PIBS system is a new, more detailed assessment method of vaginal radiation dose, which was recently proposed by the EMBRACE study and ICRU-89 document (12, 20). This point dosimetry evaluation system has many advantages compared to traditional ICRU-R point, such as the inclusion of dose points in different vaginal parts, both in the region of BT high dose and in the lower and mid vaginal points, so, it can show the dose administration along the cranio-caudal region of the vagina. Besides, this system also includes dose information of both BT and EBRT (8). In the PIBS system, the dose of PIBS series points is an important index to evaluate the vaginal dose. However, so far there is no study on the relationship between the dose of PIBS points and vaginal toxicity in pelvic radiotherapy. In this study, we observed that the dose delivered to PIBS + 2 was 108.1 ± 55.4 Gy3 for the patients with VS G ≥2, but only 90.2 ± 40.8 Gy3 with patients with VS G = 0/1. Meanwhile, doses delivered to PIBS and PIBS − 2 for patients with VS G ≥2 were also higher than those in patients with VS G = 0/1, although the differences were not statistically significant.

VRL is another important parameter for the PIBS evaluation system. It was found to be a strong risk factor for the morbidity of VS in this study. The cut-off value was set at 4.6 cm, which is the median of VRL, similar to the results of a previous dose study on many patients (10). The incidence rate of VS G ≥2 was 2.3 times higher in patients with VRL ≤4.6 cm compared to VRL >4.6 cm. We found that because of having shorter VRL, Chinese patients with CC received higher doses of radiation for total EBRT and BT in the vaginal upper two-thirds region. In univariate Cox regression, the dose of PIBS + 2 was a high-risk factor for VS. However, doses of PIBS points were not the risk factors in the further analysis using the multi-Cox regression model. A few facts can explain the results. First, PIBS points dose, especially PIBS + 2 and PIBS are significantly affected by VRL, so there is strong correlation of each other. Second, VRL in Chinese patients is shorter than that in patients of European and American. Therefore, part of vaginal that received higher dose is large. It may be a risk factor for VS. However, the PIBS point system only involves the dosimetric evaluation, so it cannot assess the dose-volume of the vagina, although, this still needs further study to verify.

Currently, the ICRU-R point is designated as a representative dose point to assess the intermediate dose for the upper portion of the vagina (13). In this study, the dose of ICRU-R point was found to be higher in VS G ≥2 group than in the G = 0/1 group. It is further confirmed that the dose of ICRU-R point is a strong risk factor for morbidity due to VS in CC patients who received radiotherapy treatment by uni- and multivariate Cox regression models. The association between dose and effect indicated that the possibility to develop VS G ≥2 is 21% with an ICRU-R point dose of 75 Gy, which is higher than the findings of the EMBRACE study (7). The reason for the difference in results may be because we used the A points planning not the three-dimensional planning.

In conclusion, doses of PIBS system points were associated with late vaginal toxicity. VRL and a dose of the combination of EBRT and BT to the ICRU-R point added to the VS risk. Physicians should pay attention to VS when the VRL of the patient is ≤4.6 cm. Besides, using a dose of ICRU-R point ≤75 Gy EQD_2_ (EBRT + BT dose) could reduce the risk of severe VS.

## Data Availability Statement

The raw data supporting the conclusions of this article will be made available by the authors, without undue reservation.

## Ethics Statement

The studies involving human participants were reviewed and approved by The First Affiliated Hospital of Xi’an Jiaotong University (No. XJTU1AF2017LSK-11). The patients/participants provided their written informed consent to participate in this study. Written informed consent was obtained from the individual(s) for the publication of any potentially identifiable images or data included in this article.

## Author Contributions

Data collection, statistical analysis, data analysis and interpretation, manuscript preparation: JW, K-sZ, and TW. Conception and design of study, data analysis and interpretation, senior reviewer: ZL. Data analysis and interpretation: R-hW and WY. Data collection and interpretation, reviewer: F-qZ, LY, Y-lW, L-cW, MS, SL, B-gL, FS, JS, Q-yZ, and JZ. All authors listed have made a substantial, direct, and intellectual contribution to the work and approved it for publication.

## Conflict of Interest

The authors declare that the research was conducted in the absence of any commercial or financial relationships that could be construed as a potential conflict of interest.

## Publisher’s Note

All claims expressed in this article are solely those of the authors and do not necessarily represent those of their affiliated organizations, or those of the publisher, the editors and the reviewers. Any product that may be evaluated in this article, or claim that may be made by its manufacturer, is not guaranteed or endorsed by the publisher.
